# Molecular Cytogenetics and Cytogenomics of Brain Diseases

**DOI:** 10.2174/138920208786241216

**Published:** 2008-11

**Authors:** I.Y Iourov, S.G Vorsanova, Y.B Yurov

**Affiliations:** 1 National Research Center of Mental Health, Russian Academy of Medical Sciences; 2 Institute of Pediatrics and Children Surgery, Rosmedtechnologii, Moscow, Russia

**Keywords:** Molecular cytogenetics, brain diseases, cytogenomics, genomic variations, genomic instability, chromosomal mosaicism, aneuploidy.

## Abstract

Molecular cytogenetics is a promising field of biomedical research that has recently revolutionized our thinking on genome structure and behavior. This is in part due to discoveries of human genomic variations and their contribution to biodiversity and disease. Since these studies were primarily targeted at variation of the genome structure, it appears apposite to cover them by molecular cytogenomics. Human brain diseases, which encompass pathogenic conditions from severe neurodegenerative diseases and major psychiatric disorders to brain tumors, are a heavy burden for the patients and their relatives. It has been suggested that most of them, if not all, are of genetic nature and several recent studies have supported the hypothesis assuming them to be associated with genomic instabilities (i.e. single-gene mutations, gross and subtle chromosome imbalances, aneuploidy). The present review is focused on the intriguing relationship between genomic instability and human brain diseases. Looking through the data, we were able to conclude that both interindividual and intercellular genomic variations could be pathogenic representing, therefore, a possible mechanism for human brain malfunctioning. Nevertheless, there are still numerous gaps in our knowledge concerning the link between genomic variations and brain diseases, which, hopefully, will be filled by forthcoming studies. In this light, the present review considers perspectives of this dynamically developing field of neurogenetics and genomics.

## INTRODUCTION

Molecular cytogenetics is defined as a specific focus of biomedical sciences targeted at studying chromosomes at molecular resolutions and at all stages of the cell cycle. It comprises a set of the techniques that operate with either the entire genome or specific DNA sequences to analyze genomic structural and behavioral variations at chromosomal or subchromosmal level [1]. One of the most appreciable insights into genomics recently offered by molecular cytogenetics (cytogenomics) during the last several years was the demonstration of exceedingly high interindividual variation of the human genome. Soon after this discovery, it has been noticed that genomic variations are an important factor for human biodiversity and disease (reviewed and summarized in [2-7]). Brain diseases are not an exception and, currently, many of them are associated with copy-number genomic variation (copy number variants or CNVs) or genomic rearrangements [7]. Moreover, the amount of CNVs, that are related to brain malfunctioning or produce susceptibility to nervous system disorders, grows dynamically [8, 9]. Therefore, the survey of interindividual genomic variations in context of human brain diseases represents an important part of current genomic research in medicine. Since all these studies were performed *via *whole genome screen and were aimed to address genomic architecture, “molecular cytogenomics”, a term merging “molecular cytogenetics” and “genomics”, appear to be apposite for covering them. Accordingly, this term will be used to describe related studies.

Genomic variations are also observed at intertissular and intercellular level, i.e. several cell populations differing with respect to their genomes are present in an organism [10]. The latter serves as a probable source for malignization [11], organ dysfunction (including the brain), and intercellular diversity [10, 12, 13]. However, these types of genomic variations are significantly less appreciated. Nevertheless, there is growing evidence for the involvement in human brain diseases [12]. This suggests reviewing of intercellular genomic variations in context of brain diseases to be of potential interest.

Here, we have attempted to compile recent data on human genomic variation and its link with human brain diseases. The present review also considers the relevance of related studies to current knowledge about the association between genomic variation and brain functioning as well as their significance for identification of genetic determinants for human brain diseases. Finally, seeing the increase of data that are actually appearing, we have tried to suggest future directions of this dynamically developing branch of genomics.

## TYPES OF GENOMIC VARIATIONS

Genomic variations are usually classified according to the resolution of techniques for their detection [14]. There are three main types of the techniques for uncovering genetic changes: cytogenetic, molecular cytogenetic and molecular genetic. Cytogenetic approaches are applied for detection of chromosome abnormalities, i.e. gross genomic variations that involve more than 3-5 Mb. Molecular cytogenetic techniques are used as for refinement of variations detected by cytogenetic analysis as for identification of subtle genomic variations at chromosomal or even subchromosomal level achieving the highest resolution of ~1 kb. Molecular genetic techniques usually operate with DNA sequences less than 1 kb, being, however, applicable for analysis of breakpoints and DNA loss/gain in cases of gross chromosomal (genomic) variations [2, 10, 14-17]. According to this classification, following types of genomic variations are distinguished:

- single base-pair modifications: point mutations, insertion/deletions, single nucleotide polymorphisms (SNPs) [5, 18, 19];
- variations affecting DNA sequences from 2 to 1000 bp: micro- and minisatellites, gene mutation (frameshift mutations, inversions), small nucleotide repeat expansions, variable number of tandem repeats or VNTRs [20-23];
- retrotransposition of mobile genomic elements: SINE and LINE insertions (0.3-10 kb) [24];
- microscopic variations (>1 kb): CNVs, segmental duplications, inversions, translocations, microdeletions, microduplications [2-7, 14];
- subchromosomal/chromosomal rearrangements: structural chromosome abnormalities (i.e. deletions, duplications, inversions, translocations) including interchromosomal tranlocations, ring chromosomes, isochromosomes [25-27];
- chromosomal heteromorphisms: variations of heterochromatic or, more rarely, euchromatic chromosome regions [28, 29];
- chromosome fragility: nonrandom breakage of specific chromosome loci [30, 31];
- supernumerary marker chromosomes: a gain of a structurally disturbed chromosome [32];
- aneuploidy and polyploidy: loss/gain of wholes chromosomes and gain of haploid chromosome sets, respectively [10, 12, 33].

Theoretically, all these types of variations can be observed both at interindividual and at intercellular level. However, a number of them (micro- and minisatellites length changes, CNVs and chromosomal heteromorphisms) have not been ever reported to produce intercellular variations of the genome. This is probably attributed to relative stability of genome regions involved in these variations during mitosis, whereas intracellular changes produced by errors in mitotic checkpoint or maintaining genome integrity are rather frequent and are consistently observed throughout ontogeny [10]. However, the existence of such intercellular genomic variations cannot be definitively excluded. Since the scope of the present review is genomic variations detectable by molecular cytogenetic or cytogenomic approaches, we shall essentially focus our attention on microscopic, subchromosomal, and chromosomal variations of the genome and their relation to brain diseases.

## GENOMIC VARIATIONS IN UNAFFECTED POPULATION

Before an association between pathogenic condition and genomic variation is made, it is first to identify the frequency of similar genome changes in unaffected population. Currently, there are numerous reports and reviews addressing CNV distribution within unaffected cohorts and over 7500 of CNVs are reported in the available literature (for more details see [2-6, 14, 19]). In average, about 12% of the human genome is covered by CNVs, which encompass diseases loci tracked from the On-line Mendelian Inheritance in Man database [19]. Consequently, even though a genomic variation is detected in an individual with a disease, its association with phenotypic features remains a matter of conjecture without extended case-control studies. For instance, a comparative analysis of structural variation in two healthy individuals reveals an immense diversification between them suggesting CNV as one of the main contributors to genetic heterogeneity in humans [34]. Therefore, to uncover pathogenic value of genome variations at microscopic and subchromosomal levels, one should test the heritability and occurrence in individuals without targeted traits [14].

Constitutional structural chromosomal abnormalities detected by cytogenetic or molecular cytogenetic techniques are not usually observed in unaffected individuals. Although structural alterations to chromosomes (balanced structural rearrangements) are found in presumably unaffected individuals, the carriers usually have reproductive problems or offspring with causative chromosome imbalances [27]. Therefore, their occurrence hallmarks clinical population. Nonetheless, small structural chromosome abnormalities such as subtelomeric rearrangements (discussed hereafter) are found to be rather common either in healthy individuals or in unaffected parents of malformed children [35, 36]. Furthermore, there is a large set of reports describing benign cytogenetically visible structural alterations to gene-containing regions of the genome or so-called “euchromatic variants”. Although these ones are frequently reported, the biomedical meaning remains uncertain suggesting them as an additional source for genome variation without apparent phenotypical effects [29]. 

Another extremely common type of chromosomal structural diversification is the variation of heterochromatic regions. It has been long noticed that pericentromeric regions of human chromosomes, short arms of acrocentric chromosomes (13-15, 21 and 22), and heterochromatic regions 1qh, 9qh, 16qh and Yqh are extremely variable in the general population [37]. Throughout the last decades, these types of genome variation have been repeatedly described following by an attempt to catalogue them [28]. Numerous researchers consider heterochromatic variations as benign morphological peculiarities of chromosomes [38]. Nevertheless, there are reports showing their higher incidence in clinical populations [28].

Chromosomal fragile sites (nonrandomly located gaps and breaks in metaphase chromosomes induced to appear by specific culture conditions) are classified as common or rare. Common fragile sites are virtually observed in all individuals, whereas rare fragile sites are those carried by less than 1 per 20 people [39]. Therefore, common fragile sites, the amount of which is about 90, represent one of the commonest types of genomic variation. Although the prevalence and expressivity of fragile sites are extremely variable, some of them (13 common and 9 rare fragile sites) are molecularly characterized and some fragile sites are associated with specific genetic diseases and genesis of chromosomal rearrangements related to cancer [30, 31].

Supernumerary marker chromosomes are a common type of chromosome abnormalities (0.043% in the general population) with extremely variable phenotypic consequences: from severe congenital malformations to the lack of abnormal phenotype [32, 40]. Supernumerary marker chromosomes are frequently observed in unaffected individuals, the reason of which is usually explained by structural peculiarities or mosaicism [41, 42]. The incidence of supernumerary marker chromosomes in unaffected individuals remains unknown.

Aneuploidy and polyploidy are generally considered as devastative conditions associated with human morbidity and mortality. However, this is attributed to aneuploidy (much more rarely, polypoidy) affecting the majority of cells of an organism or the genomic variation manifested at interindividual level [10, 12, 33]. Therefore, neither aneuploidy nor polyploidy affecting the largest proportion of cells can be observed in unaffected individuals. In contrast to interindividual genomic variations manifested as aneuploidy (polyploidy), these ones are extremely common at intercellular level and can be virtually observed in any human cell population. Aneuploid and polyploid cells are consistently observed during cytogenetic analyses. Throughout ontogeny, these intercellular genomic variations arise due to stochastic errors during mitotic cell divisions [10]. Furthermore, tissue-specific confinement of aneuploid cell lineages is observed [12, 43-45]. The latter is usually found among clinical population and is used to explain pathogenetic conditions including brain diseases [12, 44]. However, tissue-specific aneuploidization hallmarks normal prenatal development of the central nervous system [45]. To date, there is no consensus about the scale of stochastic aneuploidy (polyploidy) occurrence in unaffected individuals. Nevertheless, studies targeted at revealing the role of this type of genomic variations in human diseases as well as case-control studies always show small but significant proportion of aneuploid cells [10, 12, 45-49]. Fortunately, these genomic variations have been precisely evaluated in the brain tissue. It has been shown that up to 10% of brain cells could be aneuploid. The latter differs significantly with aneuploidy rate in the developing human brain that achieves ~35% of cells [12, 44-48, 49]. Although aneuploidy frequencies achieve large rate in the human brain and it probably contributes to neuronal diversity [12, 13], the increase of aneuploidy rate is hypothesized to be a pathogenic mechanism for brain diseases [12].

Making a conclusion of this review part, it is to note that each type of genomic variations can be detected in unaffected individuals. However, some of them manifest at intercellular level only, being associated with morbid condition affecting all cells of an organism. Subtle and gross chromosome rearrangements and heteromorphisms are almost exclusively interindividual genomic variations and have extremely diversified phenotypic effects [2-7, 10, 12, 14, 25-28, 43-51]. Table **1** addresses genomic variations detectable by molecular cytogenetic techniques in unaffected individuals. Taking a look at genomic variations in healthy individuals, it is to elucidate one major problem that is referred to definition of the pathogenic value of genomic variations. To solve this, extended studies of related genomic changes in controls or relatives of individuals with a genomic alteration are usually performed. Nevertheless, in cases of intercellular genomic variations, the increased incidence in affected tissue is likely to be related to tissue-specific malfunction.

## GENOMIC INSTABILITY AND VARIATION IN BRAIN DISEASES

Chromosome abnormalities (aneuploidy and gross structural rearrangements) were the first genomic imbalances found to be associated with human diseases [52]. Soon later, such recurrent conditions were termed “chromosomal syndromes” and, subsequently, it was estimated that together these types of genomic variation represent the leading known genetic cause of pregnancy losses and developmental disabilities/mental retardation [12, 53]. The development of molecular cytogenetic techniques allowed the uncovering of another common genetic cause of brain malfunction — subtle chromosomal imbalances. During the latest few years alone, numerous new microdeletion syndromes have been described [54]. Furthermore, there is growing amount of communications defining specific CNVs as a cause or predisposition factors for major psychiatric and neurological diseases [5, 7]. Besides this, recent molecular cytogenetic studies proposed intercellular genomic variations as an additional source for the human brain malfunction [10, 12, 44]. The aim of this part is to describe the contribution of both intercellular and interindividual genomic variations to brain diseases.

### Chromosomal Syndromes

Chromosomal syndromes can be classified according to the pattern of causative chromosome abnormality, i.e. aneuploidy, gross structural and subtle chromosome aberration. The most frequent cause of chromosomal syndromes is aneuploidy. Together it affects no less than 0.5% of newborns [12, 50, 55, 56]. Almost all these conditions are associated with different kind of brain dysfunction [12, 57-61]. Table **2** shows the contribution of common chromosomal syndromes associated with aneuploidy to brain malfunction in humans.

Both gross and subtle structural chromosome imbalances make significant contribution to brain diseases. Additionally, structural chromosome abnormalities can be the cause of recognizable syndromes, i.e. microdeletion syndromes, as well as can be found among individuals affected by presumably idiopathic mental retardation or major psychiatric and neurological disorders [10, 12, 61-65]. The main breakthrough associated with delineation of new microdeletion syndromes was related to creation of new molecular cytogenomic approaches allowing high-resolution screening of the genome based on array-CGH (comparative genomic hybridization) technology [2, 3, 65, 66-81]. However, numerous microdeletion syndromes associated with intellectual disabilities were known before the introduction of these approaches [7, 27]. To distinguish between them, we will term syndromes discovered by means of array-CGH technology as “new microdeletion syndromes” and syndromes discovered before the introduction of this technique as “old microdeletion syndromes”, though this is an arbitrary classification. Tables **3** and **4** gather the data on “new” and “old” microdeletion (deletion) syndromes associated with mental retardation (intellectual disability).

Tables **3** and **4** give only a brief overview of microdeletion syndromes excluding subtelomeric deletions (some of them are recognized as microdeletion syndromes) and recurrent rearrangements of the X chromosome. The exclusion is made because these conditions are usually associated with idiopathic mental retardation (discussed hereafter). It is also to mention subchromosomal duplications and triplications, which together significantly contribute to the etiology of brain malfunctioning (for more information see [7]). It is generally accepted, that these genomic variations are causative when manifested at interindividual level. However, there is growing amount of reports demonstrating that mosaicism involving subtle structural chromosome rearrangements (intercellular genomic variations) is involved in brain malfunctioning [10, 12]. Although the latter mainly concerns aneuploidy, a number of case reports indicates that this is valid for structural chromosome imbalances, as well [74].

As one can notice, all these chromosome imbalances are associated with brain dysfunction that is more commonly manifested as mental retardation. Additionally, it has become possible to characterize most of them, if not all, when corresponding molecular cytogenetic or cytogenemic approaches were developed. Therefore, a conclusion can be made about advancements of molecular cytogenetics providing for new genetic views on brain functioning and making direct link between numerical/structural chromosomal imbalances and mental dysfunction. 

### Mental Retardation

Mental retardation is one of the most common conditions associated with brain impairment. About 3% of the population is affected by mental retardation and about of 25% of mental retardation cases are associated with known genetic defects. Moreover, it is estimated that at least additional 25% can be associated with genetic defects, as well [65, 82]. As previously mentioned, chromosomal syndromes (including microdeletion syndromes) make significant contribution to the etiology of mental retardation. However, these are not the only genomic variations that are associated with this condition. Through development of fluorescence *in situ* hybridization (FISH) and specific DNA probes [17], it became possible to analyze subtelomeric regions of chromosomes at subchromosomal level. Because these regions are gene-rich, it has been assumed that the imbalances can lead to different pathogenic conditions including those of the brain. Initial studies have shown that up to 5% and 7% of cases of idiopathic mental retardation (moderate and severe mental retardation, respectively) are associated with subtelomeric deletions [83-85]. The latest studies have confirmed and refined this rate and have also showed that numerous subtelomeric imbalances could be benign. Current estimates show subtelomeric imbalance occurrence among mental retardation individuals (>16000) to be about 3% [35, 36, 65]. Although it was suggested that due to specifity of genomic organization of subtelomeric regions their rearrangements could be more common than those of other genomic regions, the significance of interstitial chromosome region rearrangement was not excluded. However, the lack of the possibility to screen the entire genome hindered to address this point. Introduction of array-CGH based approaches has solved the problem. Furthermore, such techniques allow screening of subtelomeric regions, as well. Recent studies have shown that together subtelomeric and interstitial subchromosomal rearrangements can be responsible for up to 10% of mental retardation cases (for review see [65]). It should be noticed that these data was obtained by an array-CGH with ~100 kb resolution. Therefore, more significant contribution of interstitial subchromosomal rearrangements involving DNA sequences less than 100 kb is hypothesized [65]. Another common cause of mental retardation is fragile X syndrome. Currently, this syndrome affects about 1% of mentally retarded individuals. It was first described as a condition associated with fragile site at Xq27.3 or FRAXA. Consequently, it was found that chromosome X fragility at this site is due to trinucleotide expansion in an X-linked gene, *FMR1* (for more details see [86]). Apart from fragile X syndrome, numerous X-linked genes are mutated in mental retardation. Moreover, X-linked mutations are a well-known source of mental handicap and are, probably, a cause of male prevalence among mentally retarded individuals. According to the latest catalogue, 215 X-linked mental retardation conditions are known and 82 genes associated with X-linked mental retardation are cloned [87]. Regardless monogenic nature of the majority of X-linked mental retardation cases, there are numerous reports indicating that genomic variations of the X chromosome detected at subchromsomal level are common. The array-CGH approaches specially elaborated for detection of the X-chromosome subtle rearrangements shows significant contribution of X-linked gene deletions and duplications to the etiology of mental retardation (4.6% in individuals more than half of which suspected of X-linked mental retardation). Current data suggests that no fewer than 7 recurrent CNVs, encompassing genes of both syndromic and non-syndromic X-linked mental retardation, are common in mentally retarded individuals (for more details see [88, 89]). Since the introduction of this array-CGH assay is at the beginning stage, more genomic variations of the X chromosome associated with mental retardation are expected [87-89].

Mental retardation is a genetically heterogeneous condition. It is assumed that numerous types of genomic variation could contribute to the etiology. Current knowledge suggests that the most significant alterations to genome in mentally retarded individuals manifest at chromosomal and subchromosomal level. Moreover, it has been recently hypothesized that intercellular genomic variations are also contributive to mental retardation [10, 12]. Therefore, further molecular cytogenetic and cytogenomic developments are intended to provide new clues on the etiology of mental retardation. Thus, it is to conclude that molecular cytogenetic approaches are indispensable to define genetic causes of mental retardation.

### Autism

Autism is one of the commonest childhood psychiatric disorders with suggested genetic background. In a previous issue of this journal, a thorough overview of chromosome abnormalities in autism has been provided [90]. This gave a comprehensive view on gross genomic variations in autism and showed the importance of their surveying for uncovering autism susceptibility genes. It was demonstrated that such genomic interindividual variations as CNVs and chromosomal microaberrations are probably associated with autism, as well [91-98]. Furthermore, it has been shown that intercellular genomic variations manifested as mosaic aneuploidy (including mosaic supernumerary marker chromsomes) are also a risk factor for autism [48]. Other types of genome variation that are found in autism are heteromorphsisms of heterochromatic regions [99, 100], gross regular and mosaic structural chromosome aberrations [101-104], and fragile sites (additionally to FRAXA (fragile X syndrome) that is common among individuals with autism) [105]. Table **5** shows recently identified genomic variations that are associated with autism.

The overview of genomic vitiations specific for autism demonstrates their vast heterogeneity. Regardless poor reproducibility of the majority of alterations to the genome, a number of microdeletions and microduplications are rather common in autism. It is also should be noted that autistic individuals frequently exhibit intercellular genomic variations. One of the commonest types is mosaic aneuploidy. It is interesting that aneuploidy manifesting as additional chromosome X in male karyotype achieves the highest rates (~10% of individuals with idiopathic autism) [48]. Taking into account the prevalence of males (the male-to-female ratio >3:1), a hypothesis linking mosaic chromosome X aneuploidy and male prevalence in autism appears intriguing [106]. Another data acquired from related studies (especially studying CNVs) is valuable for identification of autism susceptibility genes. However, although loci involved in recurrent CNVs in autism do not show positive linkage, a number of genes was demonstrated to be linked with brain dysfunction specific for this heterogeneous disease [91, 101]. Nevertheless, molecular cytogenetics and cytogenomics provide for detection of new genomic changes as well as elucidate genomic instability as a highly probable mechanism for autism pathogenesis.

### Schizophrenia

Schizophrenia is suggested to be the commonest psychiatric disorder affecting up to 1% of general population. Throughout last decades, there were brought numerous evidences for genetic background of this disease. However, recurrent mutations in schizophrenia have not been found. Nevertheless, it was shown that at least some cases of schizophrenia are associated with chromosome abnormalities [62, 64, 107-110]. Reports on structural chromosome abnormalities were used to define specific genes involved in the pathogenesis [63], whereas numerical chromosome abnormalities were assumed directly to contribute to schizophrenia or schizophrenia-like phenotype [12, 62, 64, 107, 109]. More specifically, aneuploidy was directly observed in the schizophrenia brain [44, 49]. The latter was used as a basis of neurocytogenetic theory of schizophrenia suggesting that at least some schizophrenia cases result from genomic instability (manifested as mosaic aneuploidy) in the brain [12]. Schizophrenia patients were also shown to exhibit increased levels of chromosomal fragile site expression [62, 64, 111]. Finally, several recent reports have demonstrated recurrent CNVs in schizophrenia patients [112-115]. However, other reports have not revealed significant difference between CNVs in schizophrenia and unaffected individuals [116]. Table **6** address studies of genomic variations in schizophrenia.

Studying of genomic variations in schizophrenia at high-resolution level is at the beginning stage. Therefore, it is not surprising that some studies contradict each other. It is noticeable that mosaic aneuploidy specifically affecting the schizophrenia brain appears to be an intriguing explanation of unsuccessful linkage and association studies, inasmuch as these variations of the genome are undetectable by molecular genetic techniques. Together, data on genomic variations in schizophrenia highlight genetic instabilities either at interindividual or intercellular level as a pathogenic factor for this disease as well as suggest that schizophrenia pathogenesis is probably associated with genetic instability (i.e. mosaic aneuploidy) exclusively affecting the brain. The latter can be exclusively detected by molecular cytogenetic techniques. 

### Other Neurological and Psychiatric Diseases

Molecular cytogenetic and cytogenomic studies were performed in several other neurological and psychiatric diseases. It has been shown that such common neurodegenerative diseases as Parkinson’s disease and Alzheimer’s disease can be associated with a number of genomic variations detectable at subchromsomal level [117-119]. In contrast, some other common neurological diseases (ischemic stroke) have not exhibited common specific structural genomic variations [120]. Neurodegenerative disorders of proven genetic nature have been also reported to result from microdeletions and microduplications [121, 122]. Table **7** gives a brief overview of interindividual genomic variations detectable by molecular cytogenetic or cytogenomic techniques in neurodegenerative disorders.

Microdeletions, microduplications and CNVs were also observed in bipolar disorder and childhood psychiatric disorders [8, 54, 63-65, 90, 93, 94, 112]. Gross structural chromosome abnormalities are occasionally detected in other psychiatric disorders and are exclusive in aforementioned neurological disorders [12, 62-64]. However, Alzheimer’s disease is an exception. Since neurological abnormalities of this disease resemble in some extent to those of Down’s syndrome, it was hypothesized that Alzheimer disease patients should harbor cells with additional chromosome 21. Using FISH, it has been shown that intercellular genomic variations manifesting as low-level mosaic aneuploidy of chromosome 21 are observed in fibroblasts of Alzheimer disease patients [123]. Intercellular genomic variations produced by cell cycle errors in the Alzheimer disease brain are supposed to be closely related to pathogenesis of this common devastative neurological disease (for review see [10, 12, 13]). Another neurodegenerative disease representing a valuable model of selective brain degeneration at the subtissue level is ataxia-telangiectasia. This disease is associated with chromosome instability and degeneration of the cerebellum in contrast to other brain areas. There are growing number of evidences that this disease is characterized by abnormal cell cycle events in post-mitotic brain cells, which result into intercellular genomic variations manifesting as aneuploidy, supernumerary marker chromosomes, chromosome breakage etc. These observations were used as a basis of hypothesis suggesting ataxia-telangiectasia to be an extraordinary example of chromosome instability confinement to the specific brain area (cerebellum) [124]. The latter put forward a theory hypothesizing neurodegenerative diseases association with intercellular genomic variations or genomic instability increasing in the degenerated brain areas, which are specific for a neurodegenerative disease [12, 124]. Since these intercellular genomic variations can only be addressed by molecular cytogenetic techniques, one can suggest that molecular cytogenetics is needed to uncover complex molecular and cellular pathway interaction associated with neurodegeneration.

### Brain Tumors

It has been long suggested that multilateral genomic instability lies at the origin of tumorigenesis. Genomic instabilities in human cancer can be viewed as a specific type of intercellular genomic variations. Molecular cytogenetics of cancer evidences that the commonest variations of the genome are aneuploidy/polyploidy, structural chromosome rearrangements (translocations) producing gene fusion, gene amplification, non-specific progressive variable rearrangements originating from an already rearranged karyotype [10, 11, 125, 126]. Most of these variations being increased in malignant cells produce chromosome instability, which can be viewed as a type of intercellular genomic variations, as well [10]. Chromosome instability being usually manifested as aneuploidy in malignant cell lineages is a hallmark of cancers and is used to explain numerous specific features of cancer cells [11].

Brain tumors are the second most common type of cancer in children and are associated with poor survival both in infants and adults, representing, therefore, a heavy burden for the patients and their relatives [127]. There have been growing amount of studies dedicated to detecting chromosomal imbalances and intercellular genomic variations in brain tumors. Currently, it is suggested that almost all the chromosomes are involved in aberrations associated with brain tumorigenesis. Nevertheless, there are a number of chromosomal regions that are recurrently rearranged and some oncogenes are cloned in brain tumors. Furthermore, aneuploidzation resulting in chromosome instability appear to be involved in brain tumorigenesis [128-130]. It is also to note that fragile sites are suggested to play a role in brain malignization [131]. Taking into account the meaning of molecular cytogenetic techniques for cancer research, one has to conclude that molecular cytogenetics and cytogenmics are valuable source of discoveries in brain tumor medicine.

## DETECTION OF GENOMIC VARIATIONS: A BRIEF TECHNICAL OVERVIEW

Molecular cytogenetics provides numerous possibilities to detect different types of genomic variations [15-17]. Molecular cytogenetic approaches can be arbitrarily subdivided to those providing the view of the entire genome and to those analyzing targeted genomic regions. The former is usually applied for genomic screens of chromosome or subchromosome abnormalities at different levels of resolution. The latter is useful for screening of specific chromosome rearrangements or detection of intercellular genomic variations.

Conventional cytogenetic assays are banding methods (e.g. G- or R-banding). These techniques were unique for studying karyotype for several decades of the last century leading them to become the golden standard against which all other techniques of chromosomal analysis are measured (molecular cytogenetic techniques) [15]. Standard chromosome techniques are based on cell cultivation (preparing metaphase spreads) followed by painting with specific stains (e.g. Giemsa) to produce specific banding of chromosomes. This technique allows the visualization of cellular karyotype i.e. the entire genome at cytogenetic level. FISH is one of the most applied molecular cytogenetic technique [15, 17]. However, only few FISH-based techniques provide for assessment of the whole genome (e.g. spectral karyotyping (SKY) and multicolor banding) [15]. FISH appears to be more useful for analysis of specific DNA targets [10]. Furthermore, FISH-based interphase cytogenetic assays are almost unique well-established approaches towards identification of genomic variations at all stages of cell cycle. Therefore, interphase FISH is a valuable set of techniques for uncovering intercellular genomic variations in non-cultivated cells. The ability to provide for scoring large cell populations is another advantage of FISH for these aims [10, 12, 17, 45-49, 132, 133, 134]. To get better resolution in surveying intercellular genomic variations, there are two FISH-based approaches: quantitative FISH (QFISH) and interphase chromosome-specific multicolor banding (ICS-MCB). The former is used to differentiate between FISH artifacts and true genomic variations manifested as loss of chromosomes (chromosomal regions) [135]. The latter is the unique way to analyze entire interphase chromosome structure and numbers in interphase cells [47, 136].

Conventional cytogenetic techniques do not provide for detection of genomic variations involving DNA sequences smaller than 3-5 Mb. To uncover subtle chromosome variations *via *whole genome screening, techniques based on array-CGH are more useful, because of the ability to provide for genome screening at resolution of less than 100 kb [16, 137]. The resolution of array CGH, however, depends on array platform. Additionally, there are array CGH approaches performed at single-cell level [138]. Finally, there are also several molecular genetic approaches for studying single-cell genomic variations [139, 140], that remain, unfortunately, poorly reproducible. Table **8** demonstrates the applicability of available molecular cytogenetic and cytogenomic techniques for detection of genomic variations.

Overviewing molecular cytogenetic and cytogenomic techniques, one can conclude that current biomedicine possesses state-of-art effective techniques to evaluate all types of genomic variations. However, there are numerous difficulties encountering during both introduction and application of almost all these techniques. Therefore, enhancements and modifications of existing approaches are needed for development new ones to define new genomic variations, mechanisms of the formation, and consequences of genomic variation contribution to human biodiversity and disease.

## CONCLUDING REMARKS

Molecular cytogenetics provides for studying the genome at molecular resolutions, at single-cell level, and at all stages of cell cycle. These opportunities allow uncovering numerous previously unidentified genomic variations, which together have a tremendous impact on current biomedical research. Related discoveries gave hints about the way genomic variations influence brain functioning and have elucidated new phenomena underlying brain diseases. It should be noted that molecular cytogenetic techniques are highly efficient for diagnosis of numerous diseases associated with brain dysfunction. Currently, it is estimated that from 8 to 25% of children with mental retardation and with/without additional phenotypic abnormalities require molecular cytogenetic diagnosis [10, 12, 17, 141, 142]. Therefore, molecular cytogenetics gathers indispensable tools for reliable diagnosis of brain malfunction conditions. Moreover, molecular cytogenetic and cytogenomic approaches have established new alterations to the genome that cause previously unidentified brain diseases or major psychiatric and neurological disorders (at least in a number of cases). Together, it is to conclude that molecular cytogenetics and cytogenomics will soon brought new insights into relationship between genomic variations and brain diseases.

## Figures and Tables

**Table 1:** The Occurrence of Genomic Variations Detectable by Molecular Cytogenetic (Cytogenomic) Techniques in Unaffected Individuals

Type of Genomic Variations	Interindividual Variation	Intercellular Variation	Key References
CNVs	very common^1^	unreported	[2-7, 14, 19]
Structural chromosome rearrangements detected by molecular cytogenetic techniques	very rare^2^	unreported	[36, 51]
Structural chromosome rearrangements detected by cytogenetic techniques	rare^3^	very rare	[25-27, 50]
Variation of heterochromatic regions	common^4^	unreported	[28, 37, 38, 50]
Euchromatic variants	rare	unreported	[29]
Fragile sites Common Rare	— 5	very common rare^6^	[30, 31, 39]
Supernumerary marker chromosomes	very rare	rare	[32, 40]
Aneuploidy	unreported	very common^7^	[10, 12]
Polyploidy	unreported	very common^7^	[10, 12]

1 almost in all the individuals investigated

2 single case-reports or small cohorts investigated

3 less than 1 per 1000 individuals

4 more than 1 per 1000 individuals

5 fragile sites are usually observed in a proportion of cells (metaphase spreads)

6 rare fragile are uncommonly observed in healthy individuals

7 low-level mosaics.

**Table 2:** Aneuploidy Syndromes (Common) and Brain Malfunction

Disease	Chromosome Imbalance	Brain Dysfunction	Incidence	Key Ref.
**Autosomal aneuploidy**				
Down syndrome	Trisomy of chromosome 21	Mental retardation/ neuropathological changes/conspicuous brain malformations	~1:800	[57]
Edwards syndrome	Trisomy of chromosome 18	Severe brain dysfunctions/ malformations hardly compatible with life	~1:7000	[12, 60]
Patau syndrome	Trisomy of chromosome 13	Severe brain dysfunctions/ malformations hardly compatible with life	1:6000-1:29000	[12, 60]
Trisomy 8	Trisomy of chromosome 8	Mental retardation/different morphological brain abnormalities	>100 cases reported	[12, 60]
Trisomy 9	Trisomy of chromosome 9	Mental retardation/different morphological brain abnormalities	>40 cases reported	[12, 60]
**Sex chromosome aneuploidy**				
Turner syndrome	Monosomy of chromosome X	Behavioral and cognitive disabilities/psychiatric disorders	>1:2000 (females)	[58]
Trisomy X syndrome	Trisomy of chromosome X	Severe behavioral and cognitive disabilities/psychiatric disorders	~1:1000	[12, 61]
Klinefelter syndrome	Additional chromosome X in a male karyotype	Behavioral and cognitive disabilities/psychiatric disorders	~1:500	[59]
47,XYY	Additional chromosome Y in a male karyotype	Behavioral disability (aggressive behavior)/psychiatric disorders	~1:800	[12, 61]

**Table 3:** “Old Microdeletion (Deletion) Syndromes” Associated with Mental Retardation

Deleted Region	Disease	Technique for Detection	References
1p36	Monosomy 1p36	Cytogenetic/MCG^1^/MG^2^	[66]
4p	Wolf-Hirschhorn syndrome	Cytogenetic/MCG (large deletions)	[67]
5p	Cri du chat syndrome	Cytogenetic/MCG (large deletions)	[67]
7q11.23	Williams-Beuren syndrome	MCG/MG	[27]
8q42.11-8q24.13	Langer-Giedeon syndrome	Cytogenetic/MCG^1^/MG^2^	[27]
11p11.2	Potocki-Shaffer syndrome	Cytogenetic/MCG	[68]
11q24.1	Jacobsen syndrome	Cytogenetic/MCG	[27]
15q11-q13	Angelman (maternal deletion)/Prader-Willi (paternal deletion) syndromes	Cytogenetic/MCG/MG	[69]
15q21	—	Cytogenetic/MCG	[70]
16p13.3	Rubinstein-Taybi syndrome (<50% of cases)	MCG/MG	[71]
17p13.3	Millier-Dieker syndrome	Cytogenetic/MCG/MG	[7, 27]
17p11.2	Smith-Magenis syndrome	Cytogenetic/MCG/MG	[7, 27]
22q11.2	DiGeorge syndomre	Cytogenetic/MCG/MG	[72]

1 molecular cytogenetic

2 molecular genetic.

**Table 4:** “New Microdeletion Syndromes” Associated with Mental Retardation^1^

Deleted Region	Typical Clinical Features (Apart from Mental Retardation)	References
1q41q42	Seizures, dysmorphic features, midline defects (Fryns syndrome?)	[73]
2p15p16.1	Developmental delay, short stature, microcephaly	[74]
3q29	Speech delay, autistic traits, dysmorphic features	[75]
12q14	Osteopoikilosis, short stature	[76]
15q13.3	Epilepsy, facial and digital dysmorphisms	[77]
15q24	Growth retardation, microcephaly, digital abnormalities, hypospadia, loose connective tissue	[78]
16p11.2p12.2	Distinct facial features, heart defects, short stature	[79]
17q21.31	Characteristic facial dismorphisms, hypotonia	[80]
22q11.2	Learning and behavioral problems	[81]

1 all these syndromes were discovered by array-CGH-based approaches.

**Table 5:** Genomic Variations Associated with Autism

Type of Variation	Incidence Among Autistic Individuals	Chromosomes	References
**Interindividual genomic variations**			
CNVs	~7%	almost all chromosomes (in different degree)	[91]
CNVs	10%	2p; 2q; 3p; 6p; 7p; 10q; 13q; 15q; 16p; 20p	[92]
Microdeletions (del) and Microduplications (dup)	—	16p13.1	[93]
Duplications	—	7q11.23 (Williams-Beuren syndrome region)	[94]
Microdeletions (del) and Microduplications (dup)	0.6% (del) ~1% (del+dup)	16p11.2	[95] [96]
CNVs	~7%	almost all chromosomes (in different degree)	[97]
Submicroscopic chromosome abnormalities	11.6%	2p; 2q; 3p; 3q; 5q; 7p; 7q; 8q; 10p; 11p; 12p; 13q; 14q; 15q; 16p; 16q; 17p; 18q; 19q; 20p; 20q; 21q; 22q; Xp	[98]
Heteromorphisms of heterochromatic regions	48%	1qh; 9qh; 16qh	[99, 100]
Structural gross chromosome aberrations	~5%	almost all chromosomes	[90, 101, 102]
**Intercellular genomic variations**			
Mosaic structural gross chromosome aberrations	single case-reports	3q; 20p (other chromosomes are also reported)	[103, 104]
Fragile sites (+fragile X syndrome)	—	1; 2; 3; 4; 5; 7; 9; 10; 11; 16; X	[102, 105]
Mosaic aneuploidy and supernumerary marker chromosomes	16%	9; 15; 16; 18; X	[48]

**Table 6:** Genomic Variations Associated with Schizophrenia

Genomic Variation	Brief Description (Persons, n)	References
**Interindividual genomic variations**		
CNVs	Aberrations at 4 loci containing genes encoding brain-expressed proteins (n=35)	[112]
CNVs	Gain of Xq23 (~50%) and loss of 3q13.12 (~30%) as well as frequent gains/losses in several other chromosomal regions (n=30)	[113]
CNVs	Thirteen aberrations, among them 2 were likely to be pathogenic (involving *NRXN1* and *APBA2* genes; n=93)	[114]
CNVs	Gene deletions and duplications in 15% (all the cases) and 20% (only young-onset cases) (n=150)	[115]
CNVs	No specific CNVs detected (n=260)	[116]
Microdeletion	Microdeletion of 22q11 (~1% in schizophrenia cohorts)	[72]
Heteromorphsims of heterochromatic regions	Mainly pericentric inversion of 9qh (9phqh or 9ph)	[64, 108]
Structural chromosome abnormalities	The wide spectrum of cytogenetically visible chromosome abnormalities (single case-reports)	[62-64, 107-110]
Numerical chromosome abnormalities	Sex chromosome abnormalities (1-4% in schizophrenia cohorts)	[62, 64, 107-109]
Intercellular genomic variations		
Fragile sites	Fragile sites on different chromosomes, that were rarely observed in controls	[62, 64, 111]
Aneuploidy	Mosaic aneuploidy in blood lymphocytes	[64, 109]
	Mosaic aneuploidy of chromosomes 1 (2 individuals; ~5% of cells), 18 (2 individuals; 2.5 and 0.5%), X (2 individuals; 4 and 3%) in cells of the schizophrenia brain	[44, 49]

**Table 7:** Interindividual Genomic Variations in Neurological Disorders

Disorder	Rearrangement	Gene	References
Alzheimer’s Disease	Duplication 21q21	APP	[117]
Parkinson’s Disease	Duplication 4q21 Triplication 4q21	SNCA	[118] [119]
Spinal muscular atrophy	Deletion 5q23.2	LMNB1	[121]
Charcot-Marie-Tooth (1A)	Duplication 17p12	PMP22	[122]
Ischemic stroke	Non-specific CNVs	—	[120]

**Table 8:** Molecular Cytogenetic and Cytogenomic Techniques for Detection of Genomic Variations

Techniques	Interindividual Genomic Variations	Intercellular Genomic Variations	Resolution
Banding techniques (karyotyping)^1^	+	+/-	3-5 Mb
FISH with site-specific/chromosome enumeration probes^2^^,^^3^	+	+	>1 kb
SKY^1^	+	+/-	>5 Mb
Multicolor banding (MCB)^1^^or^^2^	+	+/-	3-5 Mb
ICS-MCB^3^	-	+	3-5 Mb
BAC array CGH^1^	+	-	~1 Mb
Oligonucleotide array CGH^1^	+	-	30-50 kb
Single-cell array CGH^1^	-	+	~1 Mb
Single-cell sequencing^1^^,^^4^	-	+/-	>1 bp
Single-cell SNP array^1^^,^^4^	-	+/-	~50 kb

1 approaches studying the whole genome

2 approaches studying targeted DNA sequences or specific chromosomes

3 applicable for interphase cytogenetics and detects balanced chromosome abnormalities in interphase

4 poorly applicable for gross genomic variations.
